# Tracheobronchopathia osteochondroplastica: computed tomography, bronchoscopy and histopathological findings

**DOI:** 10.1590/0100-3984.2014.0056

**Published:** 2016

**Authors:** Gabriela Maria Ribeiro e Ribeiro, Marcelo Ricardo Canuto Natal, Eduardo Felipe Silva, Sabrina Cardoso Freitas, Waldete Cabral Moraes, Fernanda Cunha Maciel

**Affiliations:** 1 Hospital de Base do Distrito Federal (HBDF), Brasília, DF, Brazil.

*Dear Editor,*

**Figure 1:**
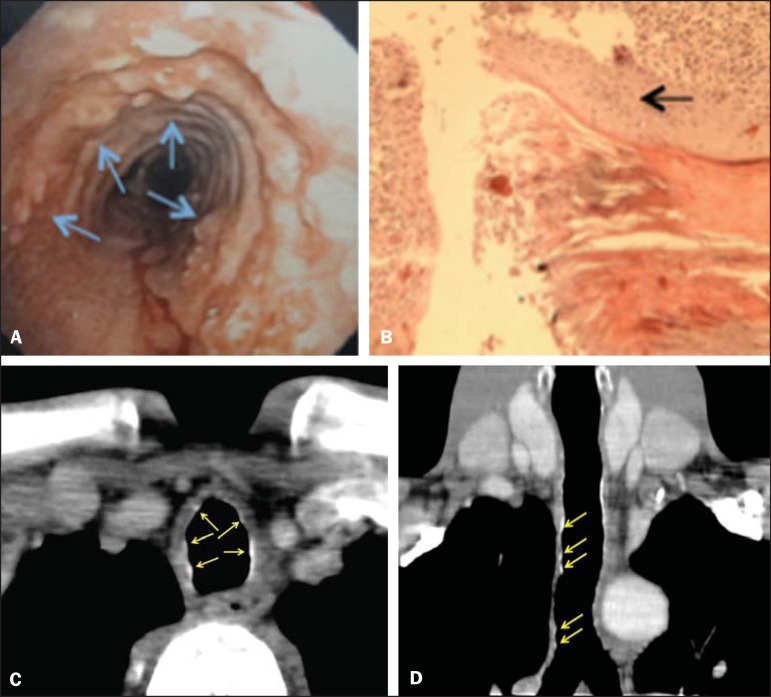
**A:** Laryngotracheobronchoscopy: whitish nodules on the anterolateral walls of the trachea (arrows). **B:** Histopathology. HE, 20×. Foci of squamous metaplasia in the trachea (arrow). **C,D:** Axial, contrastenhanced chest CT (C) and coronal reformation (D) showing micronodules, some of them calcified, on the anterolateral walls of the trachea (arrows).

A 41-year-old man with history of recurrent airways infection since his childhood, with chronic coughing and voice hoarseness for seven years. The patient was referred to undergo laryngotracheobronchoscopy that revealed the presence of whitish nodular lesions on the anterolateral walls of the trachea and at the most proximal portion of the main bronchi, whose material was sent for histopathological analysis (Figures 1A and 1B). Computed tomography (CT) showed tiny, subcentimeter, submucosal, sessile nodules, some of them calcified, at the different levels of the trachea, with predominance in the two lower thirds of the trachea, and also in the right main bronchus. No significant luminal narrowing was observed and, typically, the posterior membranous wall of the trachea was spared (Figures 1C and 1D). The patient remains under clinical follow-up with management of symptoms.

Tracheobronchopathia osteochondroplastica is a rare chronic benign disease, with male prevalence (male:female = 3:1), and predominantly manifesting between the fifth and seventh decades of life^(1,2)^. Association with several factors has been reported, as follows: chronic infections; chemical or medicamentous agents; degenerative tissue alterations; calcium and phosphorus metabolism disorders; and amyloidosis^(3,4)^.

The disease is generally asymptomatic^(1,2,5,6)^, and therefore, in most cases, the diagnosis is based on incidental findings at bronchoscopy performed to investigate other diagnoses or with therapeutic purposes, or even in series of necropsy^(1)^. In cases of symptomatic disease, cough is the main finding, present in about 66% of cases.

Generally, laryngotracheobronchoscopy raises the diagnostic suspicion and the classical finding is the presence of whitish, smooth and hard nodules, typically on the cartilaginous walls of the tracheal rings and of the proximal portions of the primary bronchi^(7,8)^.

The CT contributes to confirm the diagnosis^(4)^ on the basis of its findings, namely, thickening of the inner surface of the tracheal cartilage with irregular, sessile nodular lesions, either calcified or not, focal or diffuse, sparing the posterior (membranous) trachea and leading to luminal narrowing in the affected areas^(1,5,6,8,9)^. CT is very sensitive to detect the typical calcification of the nodules, to define the extent and distribution of tracheobronchial stenosis, as well as to characterize complications such as atelectasis, bronchiectasis, postobstructive pneumonia^(5,10)^.

Histopathological analysis shows that nodules correspond to submucosal osteocartilaginous growths. There are variable combinations of fibrotic, cartilaginous, bone, hematopoietic tissue and mineralized acellular protein matrix. The epithelium lining such nodules may be normal, or present with inflammatory or meta-plastic appearance^(5,8)^.

Some authors consider that bronchoscopic and radiological findings are sufficient to establish the diagnosis, particularly in cases where it is difficult to perform biopsy^(1,5,8)^.

The prognosis is good in most of cases and treatment only will be requested in case of complications, principally tracheal and/or bronchial stenosis^(11)^.
